# Use of Patient-Generated Health Data From Consumer-Grade Devices by Health Care Professionals in the Clinic: Systematic Review

**DOI:** 10.2196/49320

**Published:** 2024-05-31

**Authors:** Sharon Guardado, Maria Karampela, Minna Isomursu, Casandra Grundstrom

**Affiliations:** 1 Faculty of Information Technology and Electrical Engineering University of Oulu Oulu Finland; 2 Department of Computer Science Norwegian University of Science and Technology Trondheim Norway

**Keywords:** patient-generated health data, mHealth, health care professionals, mobile technologies, self-management

## Abstract

**Background:**

Mobile health (mHealth) uses mobile technologies to promote wellness and help disease management. Although mHealth solutions used in the clinical setting have typically been medical-grade devices, passive and active sensing capabilities of consumer-grade devices like smartphones and activity trackers have the potential to bridge information gaps regarding patients’ behaviors, environment, lifestyle, and other ubiquitous data. Individuals are increasingly adopting mHealth solutions, which facilitate the collection of patient-generated health data (PGHD). Health care professionals (HCPs) could potentially use these data to support care of chronic conditions. However, there is limited research on real-life experiences of HPCs using PGHD from consumer-grade mHealth solutions in the clinical context.

**Objective:**

This systematic review aims to analyze existing literature to identify how HCPs have used PGHD from consumer-grade mobile devices in the clinical setting. The objectives are to determine the types of PGHD used by HCPs, in which health conditions they use them, and to understand the motivations behind their willingness to use them.

**Methods:**

A systematic literature review was the main research method to synthesize prior research. Eligible studies were identified through comprehensive searches in health, biomedicine, and computer science databases, and a complementary hand search was performed. The search strategy was constructed iteratively based on key topics related to PGHD, HCPs, and mobile technologies. The screening process involved 2 stages. Data extraction was performed using a predefined form. The extracted data were summarized using a combination of descriptive and narrative syntheses.

**Results:**

The review included 16 studies. The studies spanned from 2015 to 2021, with a majority published in 2019 or later. Studies showed that HCPs have been reviewing PGHD through various channels, including solutions portals and patients’ devices. PGHD about patients’ behavior seem particularly useful for HCPs. Our findings suggest that PGHD are more commonly used by HCPs to treat conditions related to lifestyle, such as diabetes and obesity. Physicians were the most frequently reported users of PGHD, participating in more than 80% of the studies.

**Conclusions:**

PGHD collection through mHealth solutions has proven beneficial for patients and can also support HCPs. PGHD have been particularly useful to treat conditions related to lifestyle, such as diabetes, cardiovascular diseases, and obesity, or in domains with high levels of uncertainty, such as infertility. Integrating PGHD into clinical care poses challenges related to privacy and accessibility. Some HCPs have identified that though PGHD from consumer devices might not be perfect or completely accurate, their perceived clinical value outweighs the alternative of having no data. Despite their perceived value, our findings reveal their use in clinical practice is still scarce.

**International Registered Report Identifier (IRRID):**

RR2-10.2196/39389

## Introduction

### Background

The term “mobile health” (mHealth) has been in use for nearly 2 decades to refer to the application of mobile technologies in delivering health services and collecting data pertinent to disease diagnosis, prevention, and management [1,2]. In the last decade, the scope of mHealth has expanded to include consumer-grade devices, such as smartphones, wearable, sensors, and other quasi-medical devices, while it increasingly targets specific health conditions, in addition to wellness [2,3]. Whereas medical-grade mobile devices require clinical evidence for certification, often requiring years to bring a device to the market [4], consumer-grade mobile devices evolving at a rapid pace, and open numerous possibilities through their capacity for ubiquitous data collection [5]. mHealth solutions have become integral to many people’s lives, serving as tools for tracking health and well-being. Research has found that mHealth solutions can benefit individuals in general by fostering moderate increases in physical activity [6] or by being a convenient tool for self-management of health issues [7]. For individuals with chronic diseases, mHealth solutions have been particularly effective in offering support for condition management, goal setting, and enhancing overall satisfaction [7,8]. In addition to supporting people’s efforts to manage their health, mHealth solutions also enable the collection of electronic patient-generated health data (PGHD), which can be used in the clinical context. PGHD refer to health-related data created, recorded, and gathered by and from patients outside of the clinical settings [9,10]. PGHD encompasses a broad range of data types from both passive and active sensing [1,11]. Passive data collection usually involves sensors that are connected to a mobile device that may be worn or embedded, limiting the patient’s participation to wearing, carrying, or activating the device [12]. Active data collection requires patients to manually enter information or interact with an external device such as a peak flowmeter, glucometer, or thermometer to generate information. These data are “patient-generated” since the patient has actively participated in collecting and recording [12]. It has been hypothesized that through both passively and actively collected PGHD, health care professionals (HCPs) could gain insights into patients’ activities, lifestyle, and physical condition to inform care decisions and personalize care approaches [13].

In countries with medium or high levels of digitalization, more than 56% of people appear willing to share their personal health data, even if the purpose of sharing them is not directly related to the improvement of their health [14]. Similarly, 46.3% of individuals who owned a wearable medical device indicated having shared data with a health provider in 2019 [15]. With mHealth solutions becoming increasingly accessible, it can be expected that more people may be interested in sharing their health data with HCPs if they believe that it could help them improve health care. However, a recent study found that although providers of mHealth solutions for chronic condition self-management encourage data sharing with HCPs, few solutions are designed to facilitate HCPs’ review of these data [4]. This issue, in combination with already known challenges such as interoperability, data privacy issues, data validity, and the added burden of reviewing [9,16], makes the use of PGHD in the clinic an unrealistic possibility for many HCPs.

HCPs might have different approaches and goals when deciding to ponder PGHD collected through nonmedical mobile devices. According to Nittas et al [17], when integrating PGHD into the care process, HCPs can take the supporter or the reviewer role. In the supporter role, they limit themselves to motivating patients to use mHealth, whereas in the reviewer role, HCPs assess PGHD to complement medical data. Taking the reviewer role implies an active stance, and though some might value PGHD’s contribution to care, this type of data may still be a new and unfamiliar source of information for some HCPs [18]. For PGHD for mobile devices to be feasible as a complementary tool in the clinical setting, their use should benefit both patients and HCPs. Though the adoption of mHealth solutions by patients supports their well-being and enables the availability of PGHD, such availability does not automatically equate to usefulness for HCPs. Despite the acceptance and adoption of mHealth solutions by HCPs being one of the most influential factors regarding the success of those solutions [19,20], there has not been significant research on the role HCPs are expected to take in the use of mHealth solutions [4,17] or on the concrete experiences and motivators of those willing to review PGHD.

### Objectives

The main objective of our review is to systematically analyze existing scientific literature to identify what types of PGHD and in what health conditions HCPs have been using PGHD from consumer-grade mobile devices, as well as further context information for their motivations to use these types of data as a complementary tool in the clinic.

To attain these objectives the proposed research questions for our review are as follows: (1) In what health conditions have PGHD from consumer-grade mobile been a suitable tool for HCP? (2) What types of PGHD have HCPs found useful in the care of chronic conditions? (3) What are the main motivations behind HCPs’ decision to review PGHD from consumer-grade devices?

## Methods

### Study Design

A systematic literature review (SLR) was selected as our main research method to comprehensively synthesize evidence and prior research on HCPs’ experiences reviewing PGHD from consumer-grade mobile devices to address our research questions. We wanted to follow a transparent and systematic method to inform further research on this topic. We adopted methodologies from the Guidelines for Performing Systematic Literature Reviews in Software Engineering [21] and the PRISMA (Preferred Reporting Items for Systematic Reviews and Meta-Analyses statement) [22,23], both of which provide reliable methodologies to perform SLRs in the fields of computer science and medicine, respectively (Multimedia Appendix 1 [24]). We deemed it pertinent to combine methodological traditions from both computer science and medicine, as our research topic combines technical and care viewpoints and is interdisciplinary by nature [24].

To perform this SLR, we adhered to a systematic review protocol that was prepared before starting the searches and screening process. The protocol has been published elsewhere [25] and provides an ample description of the methods used in the search strategy and the inclusion and exclusion criteria used. The review adhered closely to the original protocol, with no significant deviations.

### Search Strategy

Eligible studies were identified through comprehensive literature searches we conducted in bibliographical databases on health and biomedicine and information technology domains. The searched databases included PubMed, ACM Digital Library (including the ACM Guide to Computing Literature), IEEE Xplore, and Scopus. The searches were carried out in May 2022.

To ensure the identification of relevant papers, we constructed the search strategy in an iterative way [25]. The search string used for each database is available in Multimedia Appendix 2. Based on the specific objectives of our review, and after conducting a pilot search in PubMed, we determined that the literature search should be constructed around 3 specific key topics: “patient-generated health data,” “health personnel,” and “mobile technologies.” We used the corresponding Medical Subject Headings (MeSH) and their possible variants to construct the final search query. Once the query had been tested, it was validated by a research librarian from the University of Oulu. After completing the electronic searches, we performed a supplementary hand search of the citations found within other SLRs and scoping reviews that were retrieved during the literature searches.

### Eligibility Criteria

The defined eligibility criteria (Table 1) aimed to include original papers that reported on the use of PGHD created via consumer-grade mHealth solutions by HCPs. PGHD reported in the studies should have been collected outside of the clinical setting, through either the patients’ use of mobile health apps or the wearable devices such as smartwatches, smart rings, fitness trackers, and similar wearable trackers; studies reporting on PGHD collected by HCPs during appointments or inside the clinical settings were excluded. Studies were limited to those involving consumer-grade devices to focus on, excluding those solely focusing on PGHD from medical-grade devices. The included papers report on the experiences of HCPs who have experience using PGHD in their clinical practice, as part of a stand-alone mHealth solution, by personal initiative, or for any other reasons. We excluded papers that focus solely on the perceptions or perspectives of HCPs as potential users of PGHD. Eligible publications were restricted to those accepted in peer-reviewed journals and conference proceedings written in the English language.

**Table 1:** Eligibility criteria.

Criteria	Inclusion	Exclusion
PGHD^a^ collection context	Outside of the clinic	In the clinical setting or during appointments with HCPs^b^
Type of mobile technologies for PGHD collection	Mobile apps and consumer-grade wearable devices	Medical-grade tracking or measuring devices
Use of PGHD by HCPs	HCPs had previously reviewed PGHD from mobile devices at the time of the study	HCPs had no previous experience reviewing PGHD from mobile devices before or during the study
Type of publication	Peer-reviewed journals and conference proceedings papers	Books, opinions, editorials, and other non–peer-reviewed sources
Language	English	Other than English

^a^PGHD: patient-generated health data.

^b^HCP: health care professional.

Though current consumer-grade mobile and wearable technologies started to become more accessible in the first half of the last decade, their impact on the health care scene started to become evident only years later. In 2013, it was acknowledged that only a few studies had assessed the impact of mobile apps in the health context, and all those studies referred to apps that had been created only for research purposes and were not available to the public at that time [26]. Therefore, we limited our search to papers with publication dates starting in 2013. Our search criteria did not delimit aspects such as the medical profile or specialties of the HCPs participating in the studies, or the health conditions treated, as we aimed to ascertain whether PGHD usage by HCPs would be more prevalent in the treatment of certain medical conditions or certain medical fields.

### Selection Process

After the electronic search, the resulting papers were imported into Covidence (Veritas Health Innovation) for screening. The screening process was divided into 2 stages carried out independently by 2 researchers (SG and MK) with computer science backgrounds and previous research experience with mHealth and PGHD. Initially, the screening was limited to titles and abstracts. Before starting this stage, the reviewers completed a joint exercise to validate the review methodology and ensure that the inclusion and exclusion criteria were correctly understood. The disagreements that arose during the initial stage were all discussed and resolved between the 2 reviewers before starting the second screening stage. The second screening round included the review of the full text of all the preliminarily included papers.

### Data Extraction

The relevant information of the included papers was collected using a structured data extraction form constructed in Covidence (Multimedia Appendix 3). The most relevant data extracted for each paper included the professions of the participants; health conditions treated; mobile technologies used; the type of PGHD collected; and the channels used for visualization. In addition, to understand what motivated HCPs to review PGHD quotes related to their motivations and conclusions related to the topic were extracted from each study.

The data extraction task was completed by the 2 original reviewers (SG and MK) and 2 additional reviewers (CG and MI), all of whom have previous research experience with the topic of this review. Each paper was randomly assigned to be examined by 2 of the reviewers. Each reviewer performed the data extraction independently. Upon completion, the data extracted by both reviewers were compared. Discrepancies were resolved through discussion between the reviewers and a final consensus was reached in all cases.

### Data Analysis and Synthesis

Quantitative and qualitative studies were included in this review. Due to the significant heterogeneity observed in the studies’ design, types of health conditions, types of PGHD, and types of mHealth solutions, methods such as meta-analysis or meta-synthesis were not deemed the most appropriate approach for the data synthesis. The extracted data were summarized using a combination of descriptive and narrative syntheses [27]. The descriptive analysis was conducted to summarize data from the different studies. This involved classifying the studies based on the type of mobile technologies used, health conditions treated, and the types of PGHD reviewed. This approach arranged the studies into more homogenous subgroups, which aided in synthesizing different types of data. The data related to the motivations of HCPs were examined using a thematic analysis, from which different categories were derived. For the narrative synthesis, similarities, and differences between the findings of different studies were identified. The analysis and synthesis comprised three major steps: (1) organization of the included studies, (2) descriptive analysis of the findings within studies, and (3) a narrative synthesis aiming at exploring interconnections between the studies.

### Quality Assessment

The quality of the included studies was assessed in parallel to the data extraction process. From the checklists, the quality of studies proposed by Kitchenham and Charters [21] were assessed. As the included studies were both qualitative and quantitative, we selected the questions that were most appropriate for our specific research questions that were present in both the qualitative and quantitative checklists.

**Figure 1:**
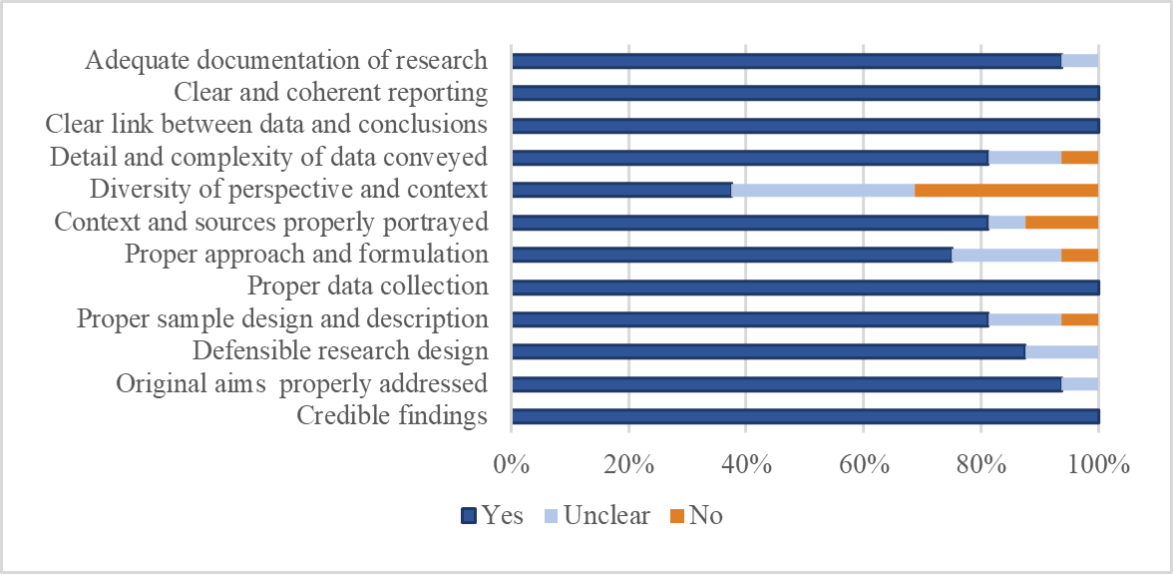
Quality assessment of included studies.

Upon assessment, all reviewers agreed that the included studies had credible findings; proper data collection methods; clear and coherent reporting; and clear links between data, interpretation, and conclusions (Figure 1).

The single topic that produced some uncertainty during the quality assessment was the lack of clarity on whether some of the selected studies had explored enough diversity of perspective and context. This can likely be attributed to the fact that almost all included studies were performed in developed countries, predominantly in the United States or Europe, which is a typical setting for digital health studies. Hence, the findings of these investigations will provide the most accurate depiction of the state of health care systems in developed countries.

### Ethical Considerations

The Ethics Committee of Human Sciences of the University of Oulu guidelines state that as no human or animal subjects were involved in the study, no separate ethics statement is required. However, the general ethical guidelines from the Finnish National Board on Research Integrity [28] guided the ethics of the study.

## Results

### Overview

**Figure 2:**
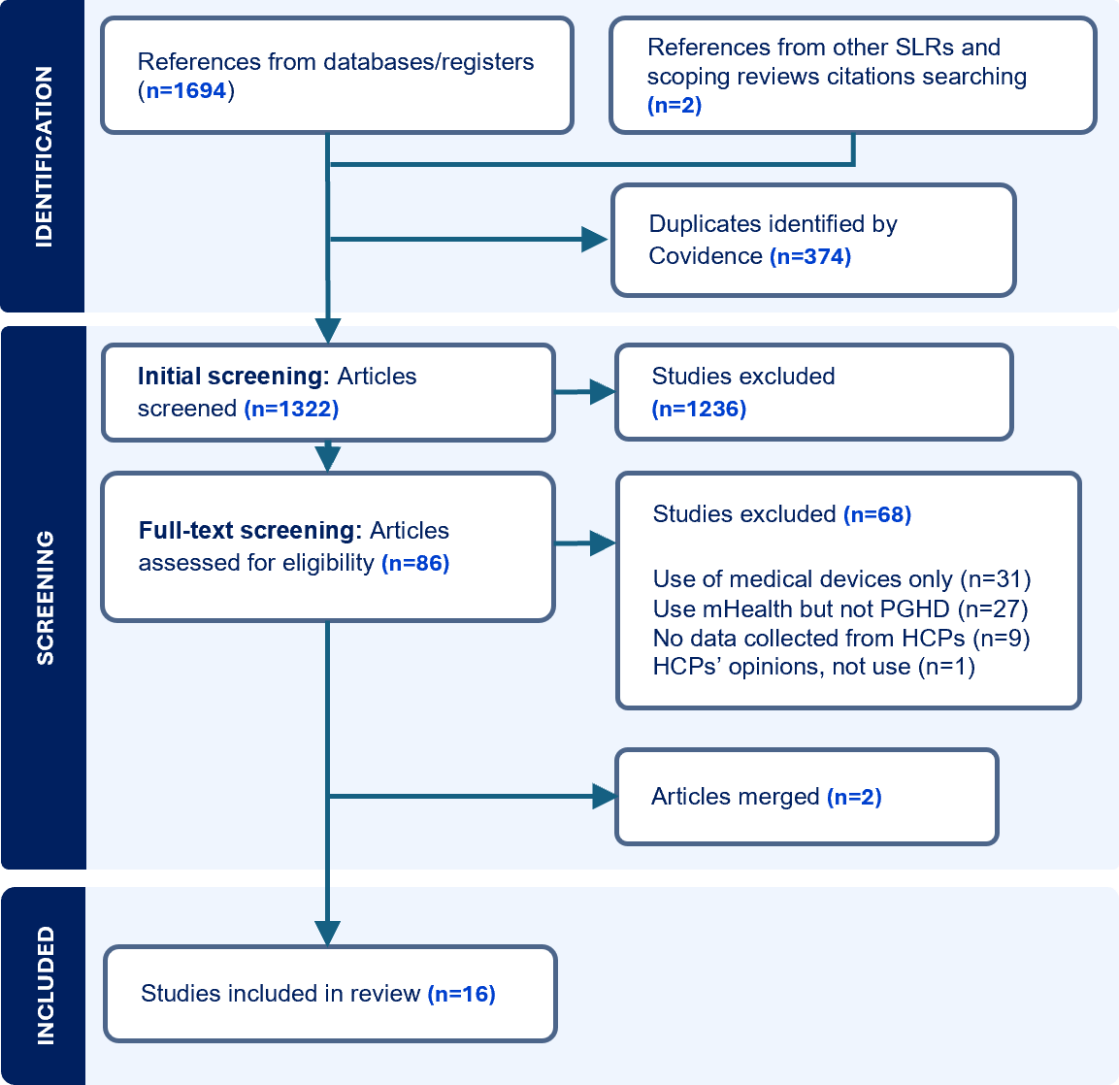
PRISMA (Preferred Reporting Items for Systematic Reviews and Meta-Analyses) flow diagram. HCP: health care professional; PGHD: patient-generated health data; SLR: systematic literature review.

Our search across electronic databases and supplementary hand searches identified 1696 papers. Covidence automatically removed 374 duplicates. We screened 1322 titles and abstracts, resulting in 86 papers for full-text screening. Following the completion of this second screening stage, 18 papers met all the inclusion criteria. However, upon closer examination, it was observed that 2 pairs of papers ([29,30] and [31,32]) had similar authors and identical samples and methodologies. Each pair was merged into a single study for analysis, resulting in the final inclusion of 16 studies for our SLR (Figure 2).

During full-text screening, papers were primarily excluded for focusing exclusively on PGHD from medical-grade devices (31/68, 46%); evaluating the usability of specific mHealth solutions, rather than PGHD use (27/68, 39%); lacking data collection from HCPs (9/68, 13%); and discussing potential rather than actual use of PGHD (1/68, 1%).

### Characteristics of the Included Studies

We included studies spanning 2015-2021. Notably, more than two-thirds of the papers (11/16, 69%) were published in 2019 or later, indicating a growing interest in the topic both before and during the COVID-19 pandemic. The predominant location was North America (11/16, 69%), specifically the United States and Canada; within Europe (3/16, 19%), Sweden and the United Kingdom were the primary locations; and 1 study was conducted in Asia and 1 in a multicountry setting. The authors used diverse methodologies for data collection, with interviews (8/16, 50%) and mixed methods (4/16, 25%) being the most common. More comprehensive insights into the specific study designs and data collection methods are available in Table 2. A complete summary of the included studies can be found in Multimedia Appendix 4 [29,30,32-46].

**Table 2:** Summary of the included studies.

References	Location	Study design	Data collection methods	Diseases treated	Participants	Type of mobile technology used for data generation	Time frame of use
Abdolkhani et al [33]	United States, United Kingdom, Australia	Qualitative study	Interviews	Diabetes, cardiac arrhythmia, and sleep disorders	Physicians, nurses, allied health practitioners	Wearable devices	Information was collected from HCPs’^a^ experiences with different wearables. The time frame was not specified
Alpert et al [29,30]	United States	Qualitative study	Interviews	Diverse conditions including musculoskeletal injuries, cardiovascular and respiratory conditions, and other nonspecific conditions	Physicians, nurses, geriatrics, physiotherapists, anesthesiology, and orthopedic surgery	Smartwatches	Information was collected from HCPs’ experiences with data PGHD^b^ from smartwatches. The time frame was not specified
Austin et al [34]	United States	Qualitative study	Surveys	Depression, substance use, blood pressure, diabetes, multiple sclerosis, cerebral palsy, perioperative care, osteoarthritis, and cognitive and gastrointestinal conditions	Physicians and researchers	Wearable devices, smartphones, medical grade wearable devices	Information was collected from HCPs’ experiences with different interventions that ranged from months to 1 week
Cerna et al [32]	Sweden	Ethnographic study	Video and audio recordings of meetings and phone calls	Pelvic cancer	Nurses	Smartphones	Patients used a bespoke mobile app between appointments. The duration of the use was agreed between the patient and the nurse
Chung et al [35]	United States	Qualitative study	Interviews	Irritable bowel syndrome and obesity	Physicians, nurses, gastroenterologists, dietitians, and behavioral psychologists	Smartphones and physical activity trackers	Information was collected from HCPs’ experiences with different solutions. The time frame was not specified
Cohen et al [36]	United States	Descriptive study	Interviews and immersion-crystallization approach	Asthma, cognitive decline, obesity, and Crohn disease	Physicians, nurses, and health coaches	Smartphones and other nonwearable devices	There were 5 different bespoke interventions, and their durations were varied
Costa Figueiredo et al [37]	United States	Qualitative study	Interviews	Infertility	Infertility specialists and endocrinologists	Smartphones	Information was collected from HCPs’ experiences with different apps. The time frame was not specified
Gupta et al [38]	Canada	Mixed methods study	Workshops and interviews	Arthritis	Physiotherapists	Fitness tracking devices (Fitbit)	The duration of the intervention was agreed upon between the patient and the physiotherapist
Holtz et al [39]	United States	Cross-sectional study	Surveys	Not specified	Physicians and nurses	Smartphones, wearable devices, web-based apps	Information was collected from HCPs’ experiences with different solutions. The time frame was not specified
Kim et al [40]	Canada	Mixed methods study	Focus groups, questionnaire	Care of older adults	Physicians, nurses and physiotherapists	Smartphones and wearable devices	Information was collected guided by a case study and from HCPs’ experiences with different solutions. The time frame was not specified
Kim et al [41]	South Korea	Field study	Interviews and observation	Diabetes, hypertension, heart disease, obesity, hyperlipidemia, sleep apnea, cancer, chronic rhinosinusitis, and myoma uteri	Physicians, internal medicine, otorhinolaryngology, family medicine, obstetrics and gynecology, and rehabilitation	Smartphones and wearable devices	Patients used a bespoke mobile app between appointments. The duration of the use is not specified
Ng et al [42]	United States	Qualitative study	Interviews	Posttraumatic stress disorder	Nurses, nutritionists, and group and individual therapists	Fitness tracking devices (Fitbit)	The patient could determine the duration of the use
Tendedez et al [43]	England	Qualitative study	Focus groups and interviews	Chronic obstructive pulmonary disease	Physicians, nurses, assistant practitioners, and physiotherapists	Smartphone	Patients used an off-the-shelf mobile app for 4 weeks
West et al [44]	United Kingdom	Qualitative study	Interviews	Not specified	Cardiologists, mental health specialists, general practitioners, heart failure and oncology nurses, emergency doctors, junior surgeons, and audiologists	Smartphones, wearable devices	Information was collected from HCPs’ experiences with different solutions. The time frame was not specified
Wu et al [45]	United States	Qualitative study	Interviews	Mental health	Psychiatrists and clinical psychologists	Smartphones, wearable devices	Information was collected from HCPs’ experiences with different solutions. The time frame was not specified
Zhu et al [46]	United States	Qualitative study	Interviews	Not specified	Physicians, physiotherapists, internist, primary care psychologist, and pediatric nephrologists	Smartphones	Information was collected from HCPs’ experiences with different apps. The time frame was not specified

^a^HCP: health care professional.

^b^PGHD: patient-generated health data.

### Medical Profiles and Specialties

Although some of the studies examined data collected from various stakeholders such as patients, researchers, hospital managers, or solution providers, our focus centered on data collected from HCPs. Collectively, the studies in our review had 355 HCPs as participants. Among the represented professions, physicians accounted for the largest number of participants, present in 81% (13/16) of the studies. While approximately half of those studies referred to physicians using a general term, the other half provided clear information about the medical specialties of the physicians. Nurses were the second most represented profession, participating in 62% (10/16) of the studies. Physiotherapists were the third most represented, participating in 38% (6/16) of the studies. Other health professions present were psychologists (3/16, 19%) and surgeons, dietitians, health coaches, and assistant practitioners, each mentioned in 12% (2/16) of the studies (Table 2).

All the studies reported the medical specialties where PGHD was being used. Those specialties included geriatrics, anesthesiology, orthopedic surgery, gastroenterology, dietetics and nutrition, behavioral and clinical psychology, psychiatry, obstetrics and gynecology, infertility, endocrinology, internal medicine, family medicine, rehabilitation, pediatric nephrology, otorhinolaryngology, and audiology.

### Health Conditions Treated

The studies examined a wide range of health conditions, classified according to the WHO *International Classification of Diseases*, *Eleventh Revision* (*ICD-11*), into categories such as endocrine, nutritional, or metabolic diseases; mental, behavioral, or neurodevelopmental disorders; diseases of the nervous, circulatory respiratory, and digestive systems, and diseases of the musculoskeletal system or connective tissue. In addition, some studies reported the use of PGHD for other types of medical tasks including perioperative care and care of older adults.

The most cited health conditions for which PGHD from mobile devices were reviewed by HCPs were diabetes and obesity, each mentioned in at least 3 studies. A quarter of the studies did not address a specific health condition. In those cases, the contextual information provided was limited to medical specialties or professions (Table 2).

### Types of mHealth Solutions

Among the 16 included studies, 5 mentioned specific mHealth solutions patients had been using to self-manage their health condition. The remaining studies mentioned commercial mHealth solutions in general. In half of the studies, HCPs reported using PGHD derived from a combination of diverse mHealth solutions, which included 1 or multiple mobile health apps and wearable devices. The remaining half of the studies addressed the experience of HCPs using PGHD exclusively generated through mobile health apps installed in patients’ smartphones (4/16, 25%) or captured from wearable devices (4/16, 25%).

### Types of PGHD

Various classifications of PGHD have been proposed in terms of purpose (self-use, behavior change, clinical use, and research), management of a condition (eg, diabetes, hypertension), data type (physiological, behavioral, or environmental), mode of data capture (using sensors, external devices, implanted devices, patient portals, web-based surveys, and manual entry), and whether the process is active, passive, or mixed [12]. In this study, we focused on classifying PGHD based on data types.

Physiological data were reviewed in all studies. In 7 of 16 studies, at least 3 different types of physiological data were collected. Weight was the most frequently mentioned physiological data, reported in 44% (7/16) of the studies, followed by mood (6/16, 38%) and vital signs (5/16, 31%). Other less commonly reviewed types of data were pain, blood glucose level, and other symptoms (Table 3).

**Table 3:** Types of PGHD^a^ used by HCPs^b^.

Classification of PGHD and type of PGHD		Studies, n
**Physiological data types**		
	Weight	7
	Mood	6
	Vital signs	5
	Symptoms	4
	Pain	3
	Glucose level	3
	Fatigue	2
	Cognition testing	2
	Menstrual history	2
	Defecation and urination frequencies	2
	Stool and urinary leakage	1
	Cough	1
	Mobility	1
	Breathlessness	1
	Stress	1
	Peak flow	1
	Sputum production and characteristics	1
	Paralinguistic	1
	Self-perception of performance (mental and physical)	1
**Behavioral data types**		
	Physical activity	12
	Eating habits	9
	Sleep	8
	Medication adherence	6
	Lifestyle	3
	Sedentariness	2
	Substance use	1
	Technology use	1
	Falls	1
	Driving	1
	Hydration	1
	Goal setting	1
**Environmental data types**		
	Location data	1
	Environmental factors	1

^a^PGHD: patient-generated health data.

^b^HCP: health care professional.

Behavioral data constituted the most used category of PGHD. More than 80% (13/16) of the studies indicated that HCPs had reviewed some form of behavioral data, although always in combination with physiological data. Physical activity seems to be the most reviewed type of PGHD produced by consumer-grade devices, with 75% of the studies reporting its use, followed by food intake (9/16, 56%), sleep quality or quantity (8/16, 50%), and medication adherence (6/16, 38%).

Only 12% (2/16) of the studies reported the use of environmental data, which were primarily collected through passive sensing, using wearables, whereas physiological and behavioral types of data were reported to be collected through either passive or active sensing or by a combination of both. For instance, certain types of PGHD, such as sleep, physical activity, or sedentariness, were collected through active sensing in some studies and through passive sensing in others.

### Access to PGHD

Diverse channels for PGHD access were presented. Notably, 19% (3/16) of the papers did not describe the precise channels HCPs used to access PGHD. Dashboards or solution portals were used in 56% (9/16) of the studies. The second most common channel was the patient’s mobile device (5/16, 31%). In a few studies, HCPs accessed PGHD through integration with the electronic health record (EHR; 2/16, 12%), by email (2/16, 12%), or from patients’ verbal summaries of data from their mobile devices (1/16, 6%).

### Motivation for Reviewing PGHD

**Figure 3:**
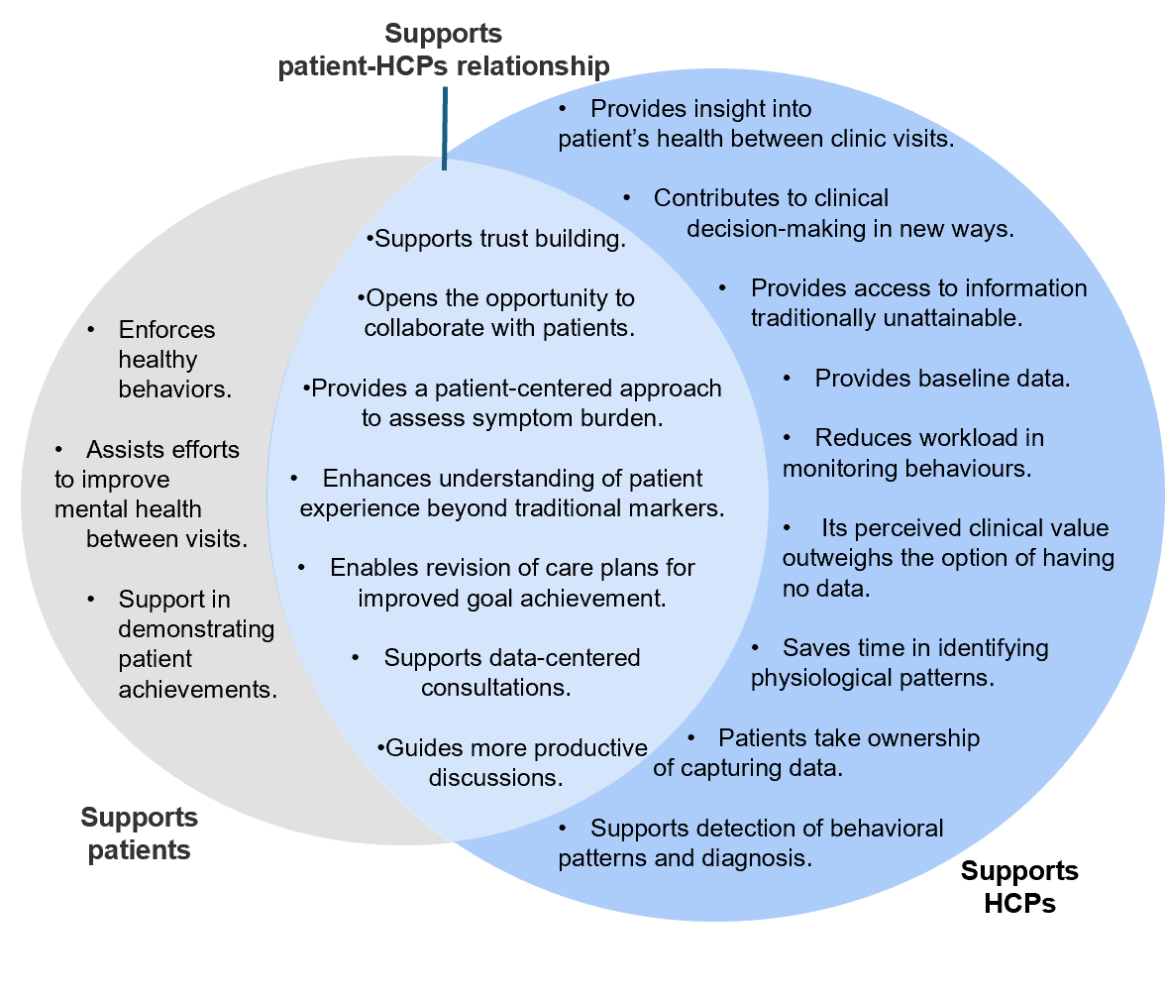
HCPs’ motivations for using patient-generated health data. HCP: health care professional.

Although not all studies cited the reasons behind HCPs’ willingness to review PGHD from consumer-grade devices, motivation for reviewing them centered into 3 main categories: benefits for the patient, supporting their clinical roles, and strengthening the patient-HCP relationship (Figure 3). Key motivations that showed how PGHD supported HCPs included topics such as accessing additional data types, identifying health patterns, and reducing data collection workload.

## Discussion

### Principal Findings

Our review underlines a growing interest in understanding the experiences of HCPs who are using PGHD in the clinic. We aimed to identify how PGHD from consumer-grade mobile devices have been used to assist them in clinical practice. HCPs, who were primarily physicians and nurses, shared their experience on the topic. The health conditions for which HCPs most resorted to PGHD were diabetes and obesity. We found that physiological data, such as weight, mood, and vital signs, and behavioral data, such as physical activity, food intake, and sleep quality, have been frequently used. HCPs had access to PGHD through different channels, such as web portals provided by the mHealth solutions or through integration with the EHR.

Previous reviews have explored the role of PGHD in facilitating prevention and health promotion [17], their use in clinical practice [18], and their effect on patient-clinician relationships [47]. However, those studies have concentrated on PGHD from medical-grade devices, which tend to be more accurate and more accepted in the medical community. PGHD created through consumer-grade mHealth solutions, although praised for their potential to transform health care, have typically not been deemed reliable or accurate enough for the clinical context [3,48,49]. Despite concerns over PGHD accuracy and reliability, HCPs recognized that their clinical value outweighs the absence of data [40]. This value comes with a caveat, as recent studies indicate that PGHD must be curated by HCPs to ascribe actionable clinical value, but even then, they can be treated as supplementary to data collected through clinically recognized standards such as through laboratory tests [50,51]. PGHD from consumer-grade solutions have been used by HCPs in the treatment of a wide variety of health conditions, although it seems common only in the care of diabetes, cardiovascular diseases, and obesity (Table 2).

The most frequently used types of data (physical activity, food intake, sleep quantity, and weight) are highly associated with lifestyle health risks, implying that access to lifestyle-related data can provide valuable insights into the control of lifestyle-related diseases. Furthermore, patients having these conditions are more willing to share PGHD, therefore, fostering HCPs’ familiarity with those types of data [15]. Our findings reveal that PGHD’s use in clinical practice remains relatively scarce [29,36,42], pointing out a gap between their potential and their current use. This finding is in line with a recent study suggesting that in comparison with the expectations of policies related to the European Health Data Space, the prompting and reviewing of PGHD from consumer-grade devices seem still relatively rare [50]. It is plausible that these types of PGHD have been used by HCPs in practice, but research on the practicalities of this phenomenon has only increased in the last 5 years.

HCPs indicated that PGHD are useful in the identification of patterns, to support certain diagnoses, and for certain types of monitoring. For example, lifestyle diseases [36], irritable bowel syndrome [42], or infertility [37] requires long-term management or presents a high level of uncertainty. In these cases, PGHD can provide longitudinal insights into patients’ health between clinic visits or even before they start treatment, saving time in identifying patterns. It is worth noting that, although HCPs in those studies acknowledged the value of PGHD, they also indicated engaging with PGHD infrequently and only with a few specific data types, in comparison with the substantial amount of data some patients want to share. For patients with chronic diseases, knowing that HCPs are reviewing their PGHD can be a comfortable way to know that they are being monitored and can provide data at the right time to facilitate decision-making and early intervention [41,52].

Multiple types of data were collected in all the studies, which signifies that as more data are collected, the need for analytical strategies that can support HCPs in reviewing and analyzing the potential relationships between different categories of data will be higher. Most existing mHealth solutions for self-monitoring lack standardized formats and mechanisms for patients to control and share PGHD [40,45]. Support for HCPs’ data access and use requires standardization and, in some cases, EHR integration [44,45].

### Limitations

We limited our inclusion to papers written in English. However, this approach may have excluded relevant papers from developing regions where English is not the primary language for scientific dissemination but where the interest and potential for mHealth solutions and PGHD are growing. Similarly, a gap in the current body of research regarding these topics in developing regions is highlighted, since all studies came from countries with high economic and digitalization levels.

PGHD is a relatively recent definition, and some relevant papers published prior to its official designation as a MeSH term may have employed alternative terminology to describe the same concept of PGHD used in our study.

The shift toward digital health solutions the COVID-19 pandemic potentiated may have modified HCPs’ perceptions of PGHD use. However, no studies explicitly examining this relationship were identified in our prior searches or a later search. Therefore, future research could explore whether the shift toward digital health has catalyzed the adoption of consumer-grade technologies and PGHD in clinical settings.

### Conclusions

Despite skepticism regarding the reliability and accuracy of PGHD and the multiple challenges that they convey, our study highlights a noticeable shift toward recognizing their practical value in health care, particularly in managing chronic conditions such as diabetes, obesity, and cardiovascular diseases. Yet, their impact in supporting the clinical practice is not clear from the literature. Many HCPs in the study, predominantly physicians and nurses, showed interest in using PGHD in the clinical workflows, albeit with a cautious approach that considers them as supplementary to traditional clinical data only. While they acknowledged the benefit of reviewing PGHD for the patient-HCP relationship, it was also noted that only certain types of PGHD are truly deemed useful and even then, they are not regularly used by HCPs. The findings call for continued research and innovation in mHealth, with a focus on enhancing the reliability, usability, and clinical relevance of PGHD, which in return can foster a culture of trust and collaboration between patients and HCPs.

## Appendix

Multimedia Appendix 1 PRISMA (Preferred Reporting Items for Systematic Reviews and Meta-Analyses) checklist.

Multimedia Appendix 2 Search strategies for all searched databases.

Multimedia Appendix 3 Data extraction form.

Multimedia Appendix 4 Summary of the included studies.

## Abbreviations

EHR: electronic health record
HCP: health care professional
mHealth: mobile health
PGHD: patient-generated health data
SLR: systematic literature review

## Data Availability

This literature review synthesizes findings from peer-reviewed journal papers and conference papers. Given the nature of this review, it does not generate new primary data; instead, it compiled and analyzed existing publications on the use of PGHD from mobile technologies by HCPs. The reviewed papers are all available in public scientific databases. The data extraction form and the extracted data are available in the Multimedia Appendix section. These resources aim to ensure the reproducibility of our methods and facilitate future research in this area.
