# Supplementary material for: Use of Patient-Generated Health Data From Consumer-Grade Devices by Health Care Professionals in the Clinic: Systematic Review
Source: J Med Internet Res. 2024 May 31;26:e49320. doi: 10.2196/49320 (PMC11179023; doi:10.2196/49320)
Supplement: Multimedia Appendix 3 [file jmir_v26i1e49320_app3.pdf]

General information

Study ID

Title

Title of paper / abstract / report that data are extracted from

Authors

Year of publication

Countries where the study was conducted

- ☐ Finland
- ☐ Denmark
- ☐ Norway
- ☐ Canada
- ☐ United States
- ☐ Australia
- ☐ UK
- ☐ Sweden
- ☐ Other

Characteristics of included studies

Aim of study / research questions/ Objectives

Main findings of the study

Most relevant result, findings or conclusions of the study.

Methods

Data Collection Methods

- ☐ Interviews
- ☐ Surveys
- ☐ Case Study
- ☐ Focus Groups
- ☐ Workshops
- ☐ Observation
- ☐ Other

Sampling Method

- ○ Snowball
- ○ Purposive
- ○ Convenience
- ○ Random Sampling
- ○ Other

Participants

Profession / Medical Specialties of participants

internal medicine, family medicine, geriatric medicine, nursing, surgery, rehabilitation, and anesthesiology

- ☐ Physicians
- ☐ Nurses
- ☐ Internal Medicine
- ☐ Geriatry
- ☐ Physiotherapists
- ☐ Gastroenterologists
- ☐ Endochinologists
- ☐ Other

Total number of participants

The number of participants in the study fit the healthcare professional description. For the SLR HCP are professional working in the provision of health services and directly interacting with patients.

Data sources

Conditions being treated

Conditions for which HCP were using PGHD as a supplementary source of information

- [ ] Diabetes
- [ ] Cancer
- [ ] Not specified
- [ ] Other

Mobile technology used for data collection

Type of consumer-grade devices being used by patients to track or generate data.

- [ ] Smartphones
- [ ] Weareable devices
- [ ] Other

Type of PGHD being used

Refers to the type of data that is being generated with the use of mobile technologies and used by HCP in care e.g. mg/dl, heart rate, body temperature, etc.

Channels for data visualization

Where are HCP able to review PGHD?

- [ ] EHR integration
- [ ] Patient device
- [ ] Patients Health Portal
- [ ] Dashboard from solution provider
- [ ] Emailed by patient
- [ ] Other

HCP main motivation to use PGHD

What motivates HCP to not only recommend eHealth solutions to their patients but also to review the data those solutions produce?
